# Multimodal mixed reality visualisation for intraoperative surgical guidance

**DOI:** 10.1007/s11548-020-02165-4

**Published:** 2020-04-24

**Authors:** João Cartucho, David Shapira, Hutan Ashrafian, Stamatia Giannarou

**Affiliations:** 1 The Hamlyn Centre for Robotic Surgery, Imperial College London, London, SW7 2AZ UK; 2 Product Development Group Zurich, ETH Zürich, 8092 Zürich, Switzerland

**Keywords:** Image-guided surgery, HoloLens, Mixed reality, Augmented reality, Computer-assisted surgery, Head-mounted display

## Abstract

**Purpose:**

In the last decade, there has been a great effort to bring mixed reality (MR) into the operating room to assist surgeons intraoperatively. However, progress towards this goal is still at an early stage. The aim of this paper is to propose a MR visualisation platform which projects multiple imaging modalities to assist intraoperative surgical guidance.

**Methodology:**

In this work, a MR visualisation platform has been developed for the Microsoft HoloLens. The platform contains three visualisation components, namely a 3D organ model, volumetric data, and tissue morphology captured with intraoperative imaging modalities. Furthermore, a set of novel interactive functionalities have been designed including scrolling through volumetric data and adjustment of the virtual objects’ transparency. A pilot user study has been conducted to evaluate the usability of the proposed platform in the operating room. The participants were allowed to interact with the visualisation components and test the different functionalities. Each surgeon answered a questionnaire on the usability of the platform and provided their feedback and suggestions.

**Results:**

The analysis of the surgeons’ scores showed that the 3D model is the most popular MR visualisation component and neurosurgery is the most relevant speciality for this platform. The majority of the surgeons found the proposed visualisation platform intuitive and would use it in their operating rooms for intraoperative surgical guidance. Our platform has several promising potential clinical applications, including vascular neurosurgery.

**Conclusion:**

The presented pilot study verified the potential of the proposed visualisation platform and its usability in the operating room. Our future work will focus on enhancing the platform by incorporating the surgeons’ suggestions and conducting extensive evaluation on a large group of surgeons.

## Introduction

Mixed reality (MR) is emerging as a vital tool in surgery as it enables the surgeon to visualise subsurface anatomical structures in 3D. This is because MR “mixes” virtual and real objects, thus allowing, for example, a surgeon to see a virtual tumour inside a real patient’s body. Besides the virtual 3D objects, MR enables a surgeon to consult a patient’s data through virtual 2D screens. Those virtual screens can display data collected before surgery (preoperatively) or during surgery (intraoperatively). The multimodal data presented on the virtual screens come from medical imaging modalities such as magnetic resonance imaging (MRI), ultrasound (US), probe-based confocal laser endomicroscopy (pCLE), and computed tomography (CT) scanners.

MR is expected to become a crucial tool for guiding a surgeon intraoperatively. The virtual objects, showing a patient’s data, can be consulted by a surgeon for decision-making and can also be moved around the operating room using mid-air hand gestures. Therefore, MR allows a surgeon to consult data when and where needed [1], making it a valuable tool for surgery. Another advantage is that using MR glasses, also known as head-mounted displays (HMDs), a surgeon can consult a patient’s data without touching real physical objects, such as a computer mouse, and therefore keeps his/her gloves sterile. Since the release of Microsoft’s (Redmond, WA, USA) HoloLens HMD, in 2016, there has been a great effort to bring mixed reality (MR) into surgery [1–11]. However, we are still on an early stage to achieve the goal of bringing MR into a standard operating room to assist surgeons intraoperatively.

Although the interest to bring MR into surgery is increasing, little attention has been paid to get feedback from surgeons participating in MR experiments. A previous work has surveyed plastic surgeons on their experience using Google Glass (Google Inc., Mountain View CA, USA) in a operating room [12]. That study has assessed the comfort level, ease of use, and image quality of Google Glass. However, Google has removed this device from the consumers market, and therefore, it can no longer be used for surgery.

A previous study suggested the use of Moverio BT-200 (Seiko Epson Corporation, Suwa, Japan) for guiding a surgery to remove a brain tumour [2]. Their study used an external optical tracking system with markers both on the HMD and next to the patient’s head. However, the advantage of using the HoloLens over other HMDs is that it is a self-contained computer that automatically tracks its position relative to the surrounding environment. Therefore, there is no need to use external cameras and markers. Additionally, since the HoloLens keeps track of its position, the virtual objects remain in a stable place even when a surgeon moves around the operating room.

Using the HoloLens, previous research has mainly focused on projecting a virtual 3D model into a patient’s body [8–10, 13] or just above it to avoid obstructing a surgeon’s line of sight [1]. The standard approach is to (1) obtain preoperative data from a patient using, for example, a CT scanner, (2) reconstruct a 3D model by segmenting the different slices of that CT scan using open-source software, such as 3DSlicer^1^ or OsiriX,^2^ (3) simplify that 3D model to contain only structures of interest for the specific surgery, and (iv) during surgery, align that simplified 3D model with the patient’s body, usually through manual registration. At this point, a surgeon can perform surgery while visualising a virtual 3D object. However, besides 3D virtual objects, few studies [9] have used MR to display surgical data through virtual 2D screens, which can also display crucial data to the surgeon and from multiple imaging modalities. Also, as the main focus of previous studies was to project a 3D model into a patient’s body, the interactions between a surgeon and the virtual world have been restricted to moving, scaling, or rotating the virtual objects.

In the present paper, we propose a MR visualisation platform to assist in intraoperative surgical guidance by projecting multiple imaging modalities simultaneously using both 2D screens and 3D virtual objects. More specifically, the platform includes a (1) 3D virtual organ model, (2) preoperative MRI patient data, and (3) images from two intraoperative imaging techniques: pCLE and iUS. Our platform extends state of the art by incorporating the following contributions:

- Two novel functionalities have been introduced to allow the surgeon to customise and interact with virtual objects, namely scrolling through volumetric data and transparency adjustment of the objects. To the best of our knowledge, none of the existing MR visualisation platforms includes these functionalities;
- A novel method has been developed to enable the communication of data from an external device (e.g. computer, imaging device) to the HoloLens for intraoperative data visualisation.

**Fig. 1:**
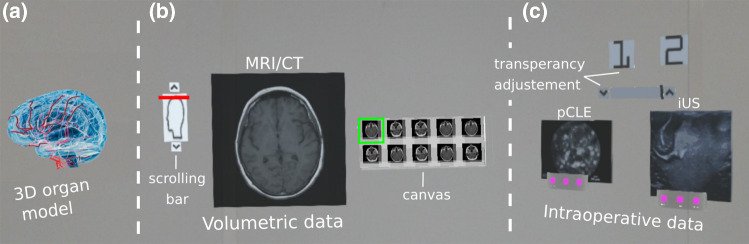
Prototype of the scene with the three MR components. These components include a 3D organ model (**a**), a volumetric imaging data component (**b**), and a component to visualise intraoperative data (**c**). Here, the volumetric data show the MRI data of a brain glioblastoma [14], and the 3D model was adopted from [15]

A pilot study has been conducted to evaluate the visualisation components and the novel functionalities and demonstrate the feasibility of using our platform for intraoperative surgical guidance. The surgeons interacted with the visualisation components and then answered a questionnaire which focuses on assessing the usability of the visualisation platform for guiding surgeons intraoperatively rather than assessing a specific device. We present the analysis of the surgeons’ scores and their suggestions for improving our platform. Finally, we discuss possible MR clinical applications of our platform for surgical guidance.

## Methodology

The platform proposed in this paper aims at providing surgeons with MR visualisation intraoperatively by integrating information from multiple imaging modalities. The MR scene consists of three visualisation components, namely a 3D organ model, an object with volumetric imaging data, and a component to visualise tissue characteristics captured using multiple intraoperative imaging probes. In this work, the volumetric data component is used to illustrate preoperative MRI/CT data, while the intraoperative data presented in the last component include probe-based confocal laser endomicroscopy (pCLE) and intraoperative ultrasound (iUS) images. An example of the MR visualisation scene as viewed from the HoloLens is presented in Fig. 1. To allow the surgeon to interact with each of the above virtual objects, the following functionalities have been developed:

- Selection of 3D anatomical structures;
- Selection of 2D slices from volumetric data;
- Repositioning of virtual objects with “Drag & Drop”;
- Scale and orientation adjustment of virtual objects;
- Scrolling through 3D volumetric data;
- Transparency adjustment of virtual objects.

To the best of our knowledge, the last two functionalities are novel, and our MR visualisation platform is the first to include them.

### Organ structure visualisation component

In our platform, a component for the visualisation of a 3D organ model has been created to display important anatomical and pathological structures selected by the surgeon such as tumours and the vasculature, segmented preoperatively, as shown in Fig. 1. The surgeon can walk around the operating room and observe the 3D model from different perspectives to obtain a better understanding of the pathology and its position in relation to important anatomical structures within the patient’s body. This component uses the following MR functionalities:

- Selection of 3D anatomical structures;
- Repositioning of virtual objects with “Drag & Drop”;
- Scale and orientation adjustment of virtual objects.

**Fig. 2:**
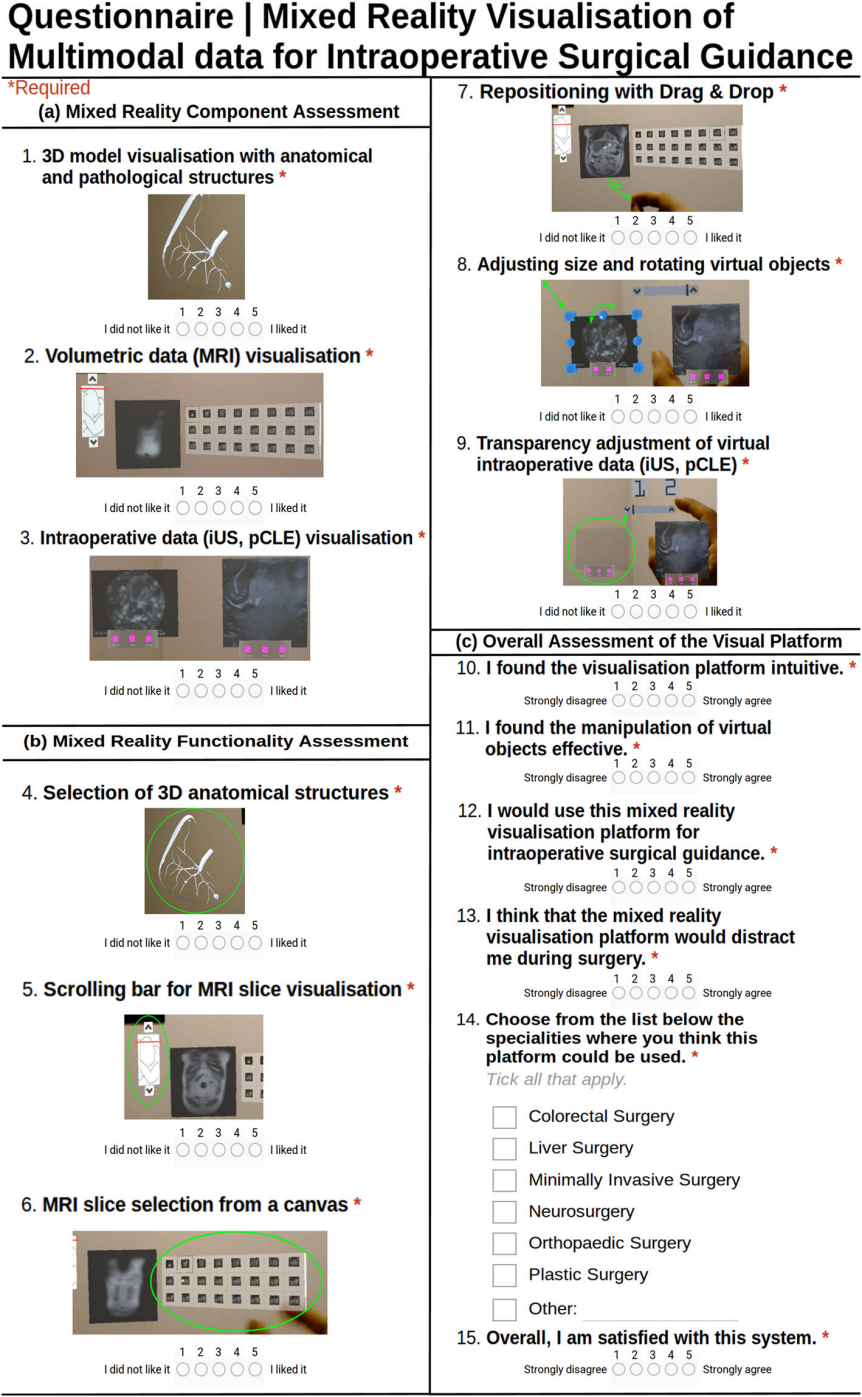
Questionnaire to assess the **a** MR components, **b** functionalities, and **c** overall assessment of our visual platform

The repositioning, scaling, and rotation of the virtual object are achieved by using two default scripts of the HoloToolkit, namely the “BoundingBoxRig” and “HandDraggable” [16]. More specifically, applying the “HandDraggable” script to a virtual object allows the surgeon to grab it and move it around following their hand movement. Upon release, the virtual object will then remain at the selected location. The “BoundingBoxRig” script provides the surgeon with a menu bar below the virtual object. Selecting “adjust” on the menu will make a bounding box appear around the virtual object with blue cubes and spheres around it, as seen in question 8 of our questionnaire (Fig. 2). The blue cubes, in the corners of the bounding box, allow the surgeon to scale the virtual object, and the blue spheres, at the edges, allow the surgeon to rotate the object.

### Volumetric data visualisation component

Another visualisation component in the proposed platform includes volumetric imaging data and has been built to allow the surgeon to access and visualise preoperative data, as shown in Fig. 1. This component uses the following MR functionalities:

- Scrolling through 3D volumetric data;
- Selection of 2D slices from volumetric data.

Using this visualisation component, the surgeon can scroll through 2D slices of volumetric data using a scrolling bar. A set of consecutive 2D slices from the 3D data around the selected organ level is presented to the surgeon inside a grey canvas (Fig. 1). The surgeon can select from the canvas a 2D slice of interest which gets enlarged to enable closer visualisation for decision-making. “Air-Tapping” on a 2D slice enlarges the selected slice and displays it in a bigger screen next to the canvas. The selected 2D slice on the canvas is highlighted with a green frame around it to indicate which sample is currently displayed in the big screen. “Air-Tapping” and then keeping the fingers pinched together and moving the hand up or down allow the surgeon to change the images displayed on the canvas and move up/down the data volume.

To enable a more precise interaction of the surgeon with the volumetric data, a scrolling bar has been placed on the left of the corresponding visualisation component, as shown in Fig. 1. More specifically, up and down buttons have been included allowing the surgeon to scroll through the 2D slices shown on the big screen, one by one. Between these buttons, a red horizontal bar is used to indicate the position of the selected slice within the 3D volume. This red bar can be used to help the surgeon localise the position of the selected slice relative to the patient’s organ. To facilitate the slice localisation even further, a depiction of the scanned organ has been added on the surface of the scrolling bar. Scrolling through volumetric data is particularly important in complex operations where the surgeon needs to constantly consult the preoperative data to estimate the location of an underlying disease with respect to critical anatomical structures or for patients with significant anatomical variations.

### Intraoperative data visualisation component

The proposed platform also includes a component for the visualisation of multimodal imaging data captured intraoperatively to facilitate tissue characterisation. As illustrated in Fig. 1, two virtual screens display tissue morphology obtained by scanning the tissue intraoperatively using pCLE and iUS imaging probes. In the scene, the buttons “1” and “2” allow the surgeon to make the corresponding virtual object appear or hide. To allow the HoloLens to receive intraoperatively data from external devices and display them on the screens, a novel server–client platform has been developed using Unity networking (UNet), the native networking project of Unity, based on the User Datagram Protocol (UDP). Our communication platform enables the transferring of data between any external device (e.g. computer, imaging device) and the HoloLens for intraoperative data visualisation. This allows any image processing technique to be applied to the data by processing it in an external computer and transferring the processed data to the HoloLens. This component uses the following MR functionalities:

- Repositioning of virtual objects with “Drag & Drop”;
- Scale and orientation adjustment of virtual objects;
- Transparency adjustment of virtual objects.

The surgeon can manipulate the virtual objects by adjusting their position and their transparency in the MR scene. The virtual object transparency can be increased or decreased using the up or down arrows, respectively, at the ends of a horizontal grey bar located below the “1” and “2” buttons (Fig. 1). The transparency is adjusted by changing the alpha value within the colour components of the virtual object. The alpha value ranges from 0.0 (totally transparent) to 1.0 (non-transparent). When the platform is initialised, the alpha parameter is equal to 1.0, but its value can change using the arrows with a step equal to 0.2. To give the surgeon an indication of the current alpha value, a black vertical line is used on the horizontal grey bar, as shown in Fig. 1. Being at the left end, the bar indicates alpha value 0.0 and being at the opposite end indicates a value of 1.0. The transparency adjustment is a clinically important functionality as it allows the surgeon to make the virtual objects fade or even completely disappear to create an unobstructed view of the surgical scene.

### Implementation details

In this work, Unity3D (Unity Technologies, San Francisco, CA, USA) version 2018.4.7f LTS was used as a game engine and Visual Studio 2017 as programming environment. In addition, the HoloToolkit 2017.4.2^3^ has been used with the HoloLens (1st Gen). The proposed MR platform has been used to visualise both brain and liver data. However, its versatile nature makes it suitable for the visualisation of any organ and for any surgical procedure. For the pilot study, we used a 3D model of a liver, adopted from GrabCAD^4^, converted to an object file (.obj) and then added to the scene in Unity. The volumetric data component was populated with MRI data from a patient with a liver hepatocellular carcinoma [14]. Since this paper aims to assess the different components and functionalities of the proposed visualisation platform, the choice of the organ does not affect the validation.

## Pilot study design

To evaluate the usability of the proposed MR platform, a pilot user study has been conducted. In total, nine surgeons participated in the study having different skill levels and different specialty backgrounds, including neurosurgery, colorectal, and bariatric surgery. The platform was presented to each surgeon, and the functionalities were explained in detail. Each surgeon was then allowed ten minutes to interact with the visualisation components and explore the different functionalities. At the end of the study, the surgeons answered the questionnaire shown in Fig. 2.

The aim of the questionnaire is to allow the participants to give their feedback and assess the usability of the proposed visualisation platform. The questionnaire consists of three parts, namely (a) mixed reality component assessment, (b) mixed reality functionality assessment, and (c) overall assessment of the visualisation platform. Most of the questions are scored in a 5-point Likert scale.

In the first part of the questionnaire, the surgeons had to score each of the three MR visualisation components described in Sect. 2, depending on their efficiency and usefulness. The second part of the questionnaire focused on the MR functionalities. In the last part of the questionnaire, the surgeons evaluated the usability of the proposed visualisation platform in the operating room. More specifically, the aim of question 12 is to investigate whether the surgeons would use our platform intraoperatively for surgical guidance. In question 14, the surgeons were asked to select all the specialties where our visualisation platform could be use, allowing multiple options to be ticked. Additionally, this question includes a “Other:” field, to enable the participants to suggest specialities which are not listed in the questionnaire. All the scores were analysed, and the results are presented in the following section.

## Experimental results

### Analysis of the questionnaire answers

In this section, we present the analysis of the collected answers we obtained. Table 1 presents both the individual and average scores for the questions where each surgeon had to rank from “1” to “5” the MR components in questions 1–3, the MR functionalities in questions 4–9, and the overall system in questions 10, 11, 12, 13, and 15. In the scoring scale, “1” corresponds to “I did not like it” and “5” to “I liked it”. From the scores, it can be observed that the surgeons’ favourite MR component is the 3D organ model, scoring a total of 4.0, followed by the preoperative volumetric data visualisation, scoring 3.8, and, finally, the intraoperative data, scoring 3.7. The most popular MR functionalities were the “Selection of 3D anatomical structures” and the “Repositioning of virtual objects with Drag & Drop”, both scoring an average of 4.1, followed by the “Resize & rotate”, scoring 3.9. From the results, we can also conclude that the majority of the surgeons found the platform intuitive and would use this platform for intraoperative surgical guidance. Also, observing the individual scores overall, it can be seen that the majority of the surgeons showed great enthusiasm for our platform.

**Table 1:** Questionnaire scores given by the surgeons

Question	Individual scores (per surgeon)									Av.
	1	2	3	4	5	6	7	8	9	score
1. 3D organ model	3	4	4	5	2	5	3	5	5	**4.0**
2. Preop. MRI	5	4	2	4	1	4	4	5	5	3.8
3. Intraop. pCLE/iUS	5	4	1	4	3	5	3	3	5	3.7
4. 3D structures selection	3	4	5	5	2	5	3	5	5	**4.1**
5. Scrolling MRI data	5	4	2	4	2	3	4	4	3	3.4
6. Select MRI slice	5	4	1	4	4	3	3	4	2	3.3
7. Drag & Drop	5	4	3	5	3	5	4	5	3	**4.1**
8. Resize & rotate	5	4	2	4	3	5	4	4	4	3.9
9. Transparency adjust.	5	4	4	4	3	5	3	3	3	3.8
10. Intuitive	5	4	4	4	2	4	3	4	3	**3.7**
11. Manipulation	5	4	4	4	3	4	4	4	3	**3.9**
12. Would use the platf.	5	4	1	3	2	5	4	3	5	**3.6**
13. Would distract me	5	4	4	2	4	2	3	3	3	3.3
15. Overall satisfaction	4	4	2	4	3	4	3	3	3	3.3
Av. score	**4.6**	**4**	2.8	**4.0**	2.6	**4.2**	3.4	**3.9**	**3.7**	

The highest scores are highlighted in bold. Note that this table does not include the answers to question 14

**Fig. 3:**
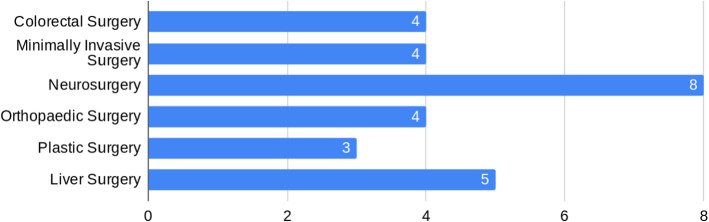
Specialties suitable for the proposed MR visualisation platform. According to the answers to question 14, eight out of the nine surgeons (89%) agree that this visual platform would be very useful in neurosurgery

The usefulness of the proposed platform to different medical specialties is investigated in question 14: “Choose from the list below the specialities where you think this platform could be used”. As shown in Fig. 3, eight out of the nine surgeons (89%) selected neurosurgery as the medical specialty which could be benefited the most from our platform. The second most suitable specialty is liver surgery, scoring 59%. Finally, “Vascular Surgery in Urology” and “Endovascular/interventional radiology” were added by the participants in the “Other:” field. In this study, our aim was to ensure we had feedback from a representative source of surgical practitioners with expertise in specialities relevant to our application. The variation in scores in this pilot study did highlight the validity of our approach that the application had varying applicability in different settings.

Compared to the visualisation options currently available in the operating theatre, our platform presents several advantages. It enables the surgeon to consult multimodal patient data intraoperatively for decision-making. Furthermore, the surgeon fully controls the data being displayed at each stage of the procedure while remaining sterile. Hence, the surgeon can manipulate the data independently, without relying on other staff of the operating theatre. In this paper, we are not trying to solve a specific clinical problem. Instead, this is an early phased demonstration of the feasibility of using our visualisation platform for intraoperative surgical guidance. In addition, independently of a surgeon’s speciality the decision-making during surgery depends on data visualisation. Accordingly, we do not assess the physical comfort, fatigue, or overall usability of the used HMD (HoloLens).

### Suggestions from the participants

In the feedback provided by the participants after they completed the study, several suggestions were made to further improve the proposed MR visualisation platform.

Regarding the visualisation components, one suggestion was to enhance the volumetric data visualisation by including all three anatomical planes (axial, coronal, and sagittal) and enabling the surgeon to scroll through each of them individually. They also suggested that it would be useful to integrate other data such as patient observations/vital signs, patient notes, and the possibility to zoom in and zoom out of the imaging data displayed.

In terms of the interaction with the different visualisation components, the incorporation of visual or auditory feedback was suggested to verify that the “Air-Tap” command has been accepted and an object has been selected. A functionality to allow the surgeon to make each of the visualisation components hide or appear as well as bring all the visualisation components into the surgeon’s direct line of vision was also recommended.

Other possible clinical applications of the proposed platform include vascular neurosurgery. For instance, it could be used to facilitate the localisation of an aneurysm in relation to the surrounding structures. The proposed platform could enable real-time localisation of a lesion (e.g. an aneurysm) in 3D relative to the surrounding important anatomical structures. The participants also indicated that the proposed platform could be useful for surgical planning, biopsy taking during stereotactic/image-guided procedures, anatomy training, and visualisation of microscopic images. It could also be integrated with a neuronavigation system, like the StealthStation.^5^ Our MR platform could also guide a neurosurgeon through the placement of an external ventricular drain (EVD) treatment of a patient with hydrocephalus. Mixed reality could guide a neurosurgeon through the placement of the EVD in real time and potentially improve a neurosurgeon’ perception of where the targeted ventricle is located, thus facilitating the overall procedure and decreasing the risk of misplacement. This system would be able to assist in avoiding damaging certain structures (i.e. blood vessels or grey matter), thus limiting collateral damage associated with the procedure, which is the crux of neurosurgery. Preliminary results were recently reported on the feasibility and accuracy of a hologram-guided EVD insertion technique [11].

Overall, this platform demonstrates the potential of harnessing the increased application of MR-based diagnostics and those of other modalities (pCLE and Ultrasound) in an integrated fashion to the wider field of precision surgery. Particularly, this work allows areas where preoperative data was informatically vital for decisions but “difficult to access” in all but a handful of high resource settings such as hybrid operating theatres. This technology is designed to offer the benefits of a hybrid theatre in visual information to every theatre space by offering the information directly to the surgeon. Specific examples include cardiac surgery where cardiac MRI images can offer surgeons the ability to reconstruct and modify valve disorders and repair and reconstruct mitral, tricuspid, and aortic valves while knowing the specific areas of myocardial risk and functionality. Spinal surgery and vertebral surgery can be enhanced by a better intraoperative appreciation of herniated discs and nerve roots. Knowledge of exact spinal imaging anatomy will guide laminar and vertebral process screw placement and operative axes. Specific cancers that are traditionally difficult to locate and are the source of multiple operative breaks to reassess images can be minimised by offering direct intraoperative tumour location, for example, neoplastic lesions in the pancreas such as cystic neoplasms or intraductal papillary mucinous neoplasms can be readily identified and managed.

This technology may prove particularly strong in the next generation of robotics that have smaller footprint and may be hand-held flexible access biomimicry-based approaches that replace the more traditional “whole theatre” robots. For example, the MRI of a prostate in augmented reality form in a large classical da Vinci system may be offered with much smaller theatre-space requirements by a surgeon operating using a hand-held robot with the same fidelity of augmented reality through their glasses at much smaller space. Furthermore, this approach can also help assess lesions that are metabolically active, where PET or other functional metabolic scans can be uploaded to guide the removal of endocrine or metabolic tumours while also offering the ability to support micro-surgical decisions to minimise unnecessary tissue removal.

### Continuing platform development

After the completion of the pilot study, taking the suggestions of the participants into consideration, we further improved our proposed visualisation platform by incorporating to it some of the surgeons’ recommendations. In particular, we added auditory feedback to the “Air-Taps” to let the surgeons know whether a command has been accepted. We also made the images on the canvas appear larger and with a higher resolution.

### Limitations of the study

There are several limitations to this pilot study. The number of surgeons who tested our platform is small, and the results may be biased towards neurosurgery since four of the participants (44%) are neurosurgeons. Future studies will be conducted with a larger number of participants, from a wider spectrum of specialities, for a more in-depth validation of the platform which will allow us to perform statistical analysis. Nevertheless, the suggestions from the surgeons are extremely valuable and expected to contribute to future research.

## Conclusions

Mixed reality is expected to become a standard visualisation tool in the operating room since it allows access to important patient data and, therefore, can facilitate surgical planning and decision-making. In this paper, we developed a successful visualisation platform which integrates multimodal imaging data for intraoperative surgical guidance. Three visualisation components have been built, and a set of novel interactive functionalities have been introduced including scrolling through volumetric data and adjusting the virtual objects’ transparency to avoid obstructing the surgeons’ view of the operating site. A pilot study has also been conducted to evaluate the usability of our platform. According to the participant’s scores and feedback, it can be concluded that neurosurgery is the medical speciality most suitable for our visualisation platform and that the 3D organ structure model is the surgeons’ favourite component. The majority of the participants found the platform intuitive and would use it in their operating room.

## Footnotes

1 https://www.slicer.org/.

2 https://www.osirix-viewer.com/.

3 https://github.com/microsoft/MixedRealityToolkit-Unity.

4 https://grabcad.com/library/liver-3.

5 https://www.medtronic.com/.
